# The role of platelet-rich plasma in repeated implantation failure: a review of biological mechanisms and clinical efficacy

**DOI:** 10.3389/frph.2026.1907558

**Published:** 2026-10-08

**Authors:** Fanourios Makrygiannakis, Thomas Vrekoussis, Maria Marmara, Aikaterini Berdiaki, Antonis Makrigiannakis

**Affiliations:** 1 Department of Obstetrics and Gynecology, Medical School, University of Crete, Herakleion, Crete, Greece; 2 Department of Histology, Medical School, University of Crete, Herakleion, Crete, Greece

**Keywords:** endometrial receptivity, molecular mechanisms, platelet-rich plasma, repeated implantation failure, reproductive outcomes

## Abstract

Repeated implantation failure (RIF) remains a significant challenge in assisted reproductive technology, with approximately two-thirds of cases attributed to impaired endometrial receptivity. This review highlights the role of autologous platelet-rich plasma (PRP) as a regenerative adjuvant for rehabilitating the uterine environment. Molecular mechanisms and clinical evidence are presented. It is evident that the concentrated growth factors in PRP—such as VEGF, PDGF, and TGF-beta—promote cell proliferation, angiogenesis, immunomodulation and tissue remodeling. *Α* consistent statistical trend is recognized toward improved clinical pregnancy and live birth rates across multiple meta-analyses. However, a critical methodological-clinical gap is identified, noting that the current evidence base is compromised by a high risk of bias, non-standardized preparation protocols, and inconsistent diagnostic criteria for RIF. While PRP shows significant biological promise in transforming a hostile uterine environment into a receptive one, its routine clinical application is currently cautioned. Until large-scale, properly blinded randomized controlled trials -utilizing standardized ESHRE definitions- are conducted, PRP should be regarded as an experimental tool rather than a validated standard of care.

## 1 Introduction

Repeated implantation failure (RIF) is a substantial hindrance for approximately 10% of couples receiving assisted reproductive technology around the world (1). For these women, achieving a clinical pregnancy has been deemed a “black box”, that often leaves clinicians with very few clear answers (2, 3). This can often cause frustration for all parties involved, along with potential financial burdens as patients navigate cycle after cycle without success (2). Therefore it is currently imperative to develop novel endometrial treatments in order to combat these lasting reproductive challenges (4). There is a great variety of upon RIF definition across the literature (5). It is generally defined as the failure to achieve a clinical pregnancy after a specified number of high-quality embryo transfers, with varying thresholds for the number of transferred embryos and cycles (6). The lack of consensus further complicates the proper diagnosis of patients, and the objective evaluation of adjuvant treatments becomes difficult (7). Current trends in the field are moving from number-based definitions to a probabilistic approach that incorporates cumulative success, maternal age, and embryo genetics (7, 8). It is being realized that many patients formerly diagnosed with RIF may reflect random biological variation, and that a standardized definition needs to be established to provide a clear indication of implantation failure to provide a clear pathway for therapy.

Increasing evidence suggest platelet-rich plasma (PRP) for endometrial rehabilitation. PRP is a an autologous concentrate of platelets with platelet counts over the normal physiological level prepared from whole blood of patients for use in regenerative medicine (9). Within the alpha-granules of platelets are high concentrations of growth factors such as VEGF, PDGF, and TGF-beta (4). Upon intrauterine infusion, the platelet growth factors are thought to improve tissue regeneration, improve vascularity, and restore the endometrial “biosensor” function (1, 7).

This review provides a holistic approach by integrating biological mechanisms and the evaluation of clinical evidence to bridge the gap of endometrial receptivity challenges in reproductive medicine.

## 2 Methods

The current work is a narrative review. The Pubmed and the Google Scholar database was searched in April 2026 using the key-words “Platelet-rich Plasma” or “PRP” combined with the term “Implantation failure”. No time-point restriction was applied. The search included meta-analyses, systematic and narrative reviews. All retrieved articles were assessed and their references were selectively retrieved. All decisions were made by the authors with consensus. A final search prior submission was performed in June 2026 to ensure that the presented data was up-to-date.

## 3 PRP products: definitions and classification

### 3.1 Definition

PRP is defined as an autologous volume of plasma with a concentration of platelets that is higher than baseline platelet counts found in whole blood (10, 11). PRP product presents a typical platelet concentration that is two- to five-fold higher than that of whole blood (12). Despite the fact that a minimum platelet concentration range of 1.0–1.9 × 10^6^ platelets/µL has been used to ensure a sufficient reservoir of signaling molecules (13, 14), evidence supports a ceiling effect (1.5 × 10^6^ platelets/*μ*L) above which further effect is not observed (15).

### 3.2 PRP preparation

PRP preparation begins with the blood collection using anticoagulants, and continues further with platelet isolation via centrifugation. The choice of anticoagulant during blood collection is important for PRP preparation. Citrate-based formulations, such as sodium citrate and Acid Citrate Dextrose Solution A (ACD-A), serve as the clinical standards. They function via reversible calcium chelation to prevent the coagulation cascade and suppress premature platelet activation during centrifugation protocols (16, 17). Conversely, agents like EDTA compromise cell membrane morphology, while heparin induces premature degranulation and confounding platelet aggregation (16, 17). For clinical application, residual citrate lowers the local pH, which can induce microenvironmental tissue irritation and transiently inhibit angiogenesis. Consequently, exogenous calcium restitution is often applied immediately before infiltration to neutralize chelation, buffer the pH, and restore full regenerative potency (18). In order to increase concentration and recovery, the double-centrifugation methods are performed in two phases: a low-speed soft spin followed by a high-speed hard spin (13, 19). According to technical parameters, a soft and hard spin performed at 300× g and 700× g, respectively, allows for robust separation with minimal platelet loss. This method has demonstrated a significant increase of platelet concentration in comparison to whole blood (13, 19). The hard spin is an essential step for concentrating platelets into a small final volume, creating a high reservoir of growth factors.

PRP can be applied either in its inactive liquid form or in its activated gel form (20). The inactivated form, when administered exerts a gradual long-term effect, since activation is progressively achieved *in situ*, with the growth factors being constantly released in low concentrations. However, the maximum PRP effect is exerted via an initial platelet activation being achieved either by chemical (thrombin, thrombin receptor agonist peptide-TRAP, calcium salts, collagen and ADP) or mechanical (freeze-thaw cycles, sonication) means (21). In that case, a burst of growth factors is immediately released. Although mainstream approach involves platelet activation (22), both forms may present benefit (20): the liquid form is uniformly distributed exerting a diffuse effect, while the activated gel form may be more locally applied and therefore anchored towards a sustainable endometrial repair.

### 3.3 PRP classification

In light of the heterogeneity of preparation protocols, a classification was previously suggested (10) based on technical processing, cellular content, and fibrin density, dividing all autologous platelet products into four families: Pure Platelet-Rich Plasma (P-PRP), Leukocyte-Rich PRP (L-PRP), Pure Platelet-Rich Fibrin (P-PRF), and Leukocyte-Rich PRF (L-PRF) (10, 23). By using these four terms and replacing all the non-standardized commercial denominations, one can overcome the poor reporting quality found in the literature (15, 23). While all formulations contain elevated platelet concentrations, Pure PRP (P-PRP) and Leukocyte- and Platelet-Rich Plasma (L-PRP) serve distinct clinical functions based on their white blood cell counts. P-PRP features minimal white blood cells, enabling a rapid release of key growth factors with minimal inflammatory risk, making it the preferred choice to enhance endometrial receptivity and rapid cell proliferation in IVF patients (12, 24–26). Conversely, L-PRP incorporates high levels of leukocytes that trigger a stronger local inflammatory response to drive immune modulation and tissue remodeling, which is typically reserved for managing chronic inflammation or severe fibrosis (12, 24–26). Moving from the fluid to the solid phase stable biomaterials, the platelet products belonging to the second generation are called Platelet-Rich Fibrin (PRF). Two main families can be discriminated: L-PRF and P-PRF. These products are characterized by a high-density 3D fibrin network,formed without the use of anticoagulants (15, 27, 28). This fibrin architecture ensures a release of growth factors, such as VEGF and TGF-beta, for up to two weeks (10, 27). In reproductive physiology, this platelet-rich fibrin matrix could be a valid tool to address severe endometrial injuries, such as Asherman's syndrome. Compared to the liquid variant, the fibrin network of PRF has a different size of the fibers and an enhanced resistance to degradation, contributing to the reconstruction of the endometrium (15, 27, 29).

Since this initial classification, several approaches have been proposed, incorporating all the relevant information upon preparation and activation; of note the last two thorough approaches that take into account among others platelet concentration, white blood and red blood cell presence and the mode of activation (30, 31).

## 4 PRP composition

PRP consists of platelets, along with leukocytes, residual red blood cells and plasma. Platelets carry an extensive repertoire of bioactive molecules, organized in several components namely: *α*-granules, dense granules, lysosomes and platelet-derived exosomes.

### 4.1 Platelets

#### 4.1.1 Alpha granules

##### 4.1.1.1 a growth factors

The regenerative capacity of PRP in the treatment of repeated implantation failure is mainly encoded in alpha-granules. Constituting 10% of total platelet volume, these are specialized storage sites (9). Alpha granules act as a large reservoir of sequestered growth factors, primarily VEGF, PDGF, and TGF-beta (32–34), driving endometrial receptivity and sub-endometrial angiogenesis in RIF patients (13, 14, 35). Additionally, the ensuing secretome acts as a mediator of coagulation and tissue healing (9, 36). Concentrated platelets in PRP constitute an autologous depot of these molecules, where they are stored until formal platelet activation triggers their release (36). In that view, signaling factors are only delivered to the endometrium when a biochemical trigger (calcium or thrombin) induces degranulation (13).

Specific growth factors within alpha-granules act on distinct physiological processes, driving endometrial receptivity. For instance, VEGF supports neoangiogenesis and endothelial mitogenesis, leading to the improvement of subendometrial blood flow and counteracting hypoxia in patients with thin endometrium (9, 37). On the other hand, PDGF stimulates the chemotactic migration and proliferation of endometrial stromal cells, promoting functional wound healing (29, 38, 39). Finally, TGF-beta acts in the regulation of collagen synthesis and matrix remodeling, preventing pathological fibrosis and scarring of the basal layer (9). These growth factors act synergistically to modify the hostile uterine environment, creating an adequate site for embryo implantation and maintenance of pregnancy (29, 40, 41).

The selection of PRP administration timing is determined by the distinct release kinetics of various growth factors. TGF-beta and FGF are released in a quick burst within the first hour after activation, with levels declining rapidly thereafter (37). On the other hand, PDGF and VEGF remain detectable over a seven-day period (37). The method of activation influences these kinetics, since thrombin-activated platelets degranulate rapidly, whereas calcium-only activation achieves a more endogenous release profile (13, 36, 42). Interestingly, calcium activation may be preferred to maximize the immediate concentrations of PDGF-BB (43). Although the yield of PDGF-BB is impacted by the activation method, the level of TGF-beta1 remains consistent across all activation groups using both chemical and mechanical triggers (43). Furthermore, fibrin clot formation allows the entrapment of growth factors and their slow, week-long, release onto the endometrium (13). The lifespan of activated PRP lasts approximately one week, and concentrations begin to decline after 72 h (36). This prolonged availability is crucial for supporting the multiple sequential stages required for the implantation cascade.

##### 4.1.1.2 b cytokines and chemokines in alpha-granules

The therapeutic mechanism of Platelet-Rich Plasma in repeated implantation failure involves platelet secretome with growth factors, cytokines and chemokines to direct tissue regeneration (13). PRP releases significantly higher concentrations of the anti-inflammatory cytokines IL-4 and IL-13 compared to whole blood (13). RANTES (CCL5) and IL-12 are also demonstrating significant differences between PRP and whole blood, with PRP containing higher RANTES and IL-12 concentrations (44).

The chemokine gradient, in which RANTES plays a role, induces migration of stem cells and uNK cells to the site of injury (45, 46) by partly mediating the adherence of monocytes to endothelial cells and subsequently acting as a strong chemoattractant (44). To this end, PRP-induced chemoattraction secures the migration of progenitor cell populations that establish the transition of inflammation to tissue repair and resolution (46).

#### 4.1.2 Dense granules, lysosomes and platelet-derived exosomes

These three components have not been studied in human reproduction. However, extrapolation from other areas of PRP administration is suggestive of an auxiliary role. In that view, dense granules deliver an array of small molecules (ADP, ATP, serotonin, calcium, histamine epinephrine and polyphosphates) promoting platelet activation and immune signaling (9, 12). Lysosomes release acid hydrolases and antimicrobial proteases contributing to ECM remodeling and antimicrobial defense (9, 12). Finally, platelet-derived exosomes may deliver encapsulated growth factors, mRNA and miRNA molecules, enhancing intercellular communication and regenerative signals (12).

### 4.2 Leukocytes

Leukocytes are important to PRP but may have a dual and opposing effect on tissue regeneration; high concentrations of neutrophils may be detrimental to regeneration and promote catabolic inflammatory cytokines (13, 23), whereas monocytes and macrophages promote the M1-M2 macrophage polarity that benefits the healing process by removing debris and promoting regeneration (23). However it must be highlighted that the cytokine profile in the final PRP product varies, depending on the donor, as the initial individual adaptive immune response to injury translates into the leukocyte composition in the PRP preparation (47).

### 4.3 Residual red blood cells

Residual red blood cells are considered a negative effect in any PRP application, since their degradation is associated to oxidative stress and subsequently to tissue damage (12). This is the reason for maximizing the effort to omit red blood cells from the final concentrate.

### 4.4 Plasma

Plasma, the fluid matrix of blood, is a dynamic reservoir of biomolecules reflecting systemic health. Beyond well-characterized proteins like albumin, immunoglobulins, and coagulation factors, proteomic studies have identified over 1,500 plasma proteins (12). However, the role of plasma has not been studied in the context as a component of PRP in human reproduction.

## 5 PRP mechanisms of action on endometrial receptivity

While clinical outcomes provide the statistical justification for Platelet-Rich Plasma in reproductive medicine, understanding the specific biological pathways is essential to move the therapy beyond an experimental status. Within the framework of this Review Paper, Platelet-Rich Plasma is defined not as a single-target drug, but as a complex bioregulator that simultaneously influences cell growth, blood flow, and immune signaling as shown in Figure 1. This mechanistic logic explains how the targeted release of growth factors triggers a biological cascade that transforms a hostile or thin endometrium into a functional, receptive tissue.

**Figure 1:**
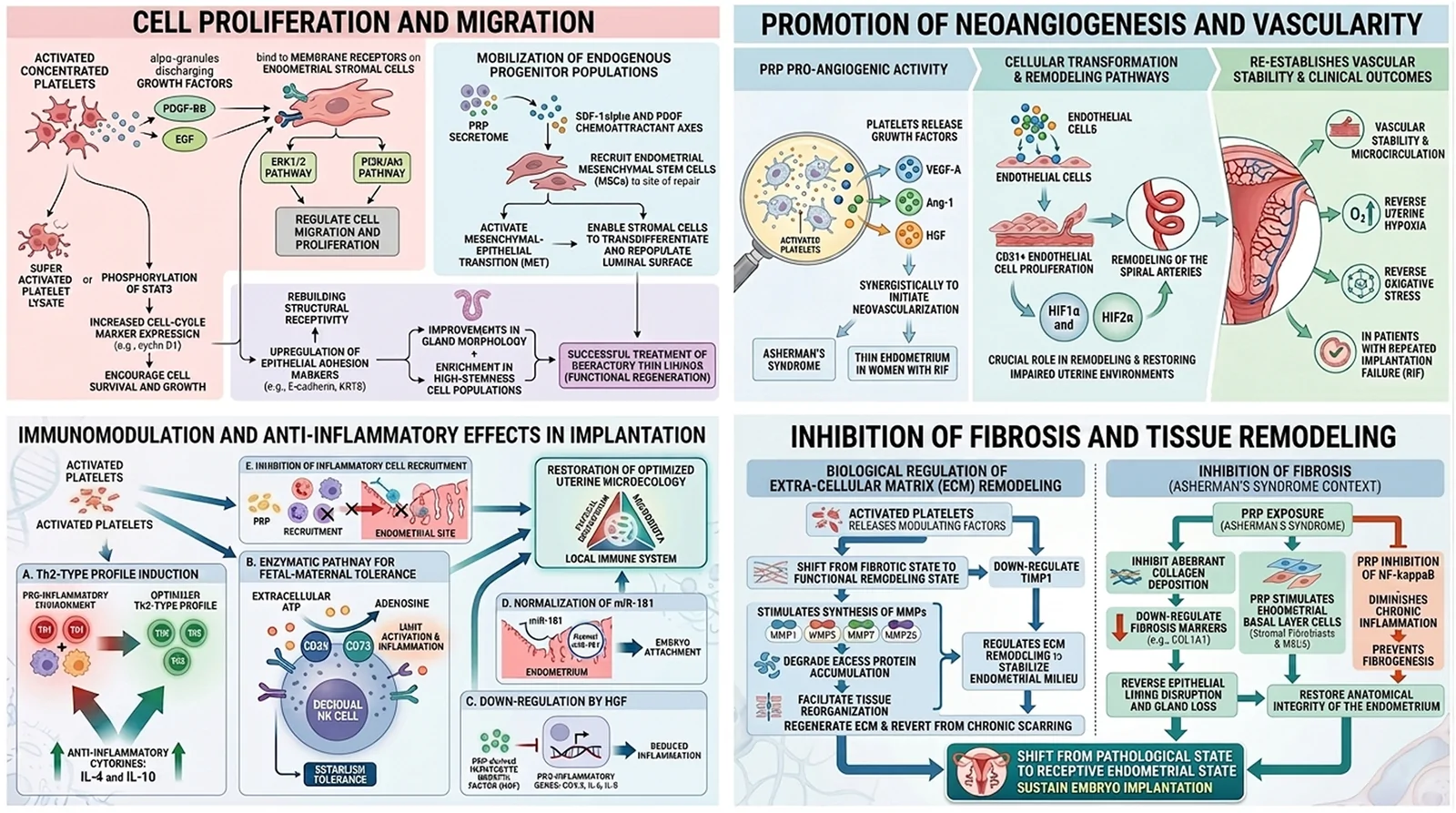
Multimodal mechanisms of PRP on endometrial receptivity. Schematic representation of the four biological pillars through which Platelet-Rich Plasma modulates the uterine environment. The mechanisms are interdependent and collectively contribute to functional regeneration. PRP acts as a concentrated reservoir of biological mediators. (Created by the Google Gemini Nano Banana 2 AI tool by improving an initial author-made version).

### 5.1 Enhancement of cell proliferation and migration

As described in previous sections herein, when concentrated platelets are activated, their alpha-granules discharge growth factors and initiate regeneration cascades (9). Once released in the uterine cavity, these growth factors—including PDGF-BB and EGF—bind to membrane receptors on endometrial stromal cells, activating mitogenic pathways such as ERK1/2 and PI3 K/Akt, to regulate cell migration and proliferation (48–50). Concurrently, evidence from an animal model for studying thin endometrium suggests that super activated platelet lysate leads to phosphorylation of STAT3 and increased cell-cycle marker expression, such as cyclin D1, encouraging cell survival and growth (9, 51).

Furthermore, the ability of PRP to treat repeated implantation failure also relies on mobilization of endogenous progenitor populations. In case of treating thin endometrium—a potential cause of RIF—it was shown that through SDF-1alpha and PDGF chemoattractant axes, the secretome of PRP recruits endometrial mesenchymal stem cells to the site of repair (9, 52). These cells, in turn, activate the mesenchymal-epithelial transition (MET), enabling stromal cells to transdifferentiate and repopulate the luminal surface (52). Moreover, the upregulation of epithelial adhesion markers, such as E-cadherin and KRT8, are important for rebuilding structural receptivity (52). In agreement with this, histology scores showed improvements in gland morphology and enrichment in high-stemness cell populations. These features are important for the successful treatment of refractory thin linings, as this represents functional regeneration as opposed to simple hypertrophy (51, 52).

### 5.2 Promotion of neoangiogenesis and vascularity

The biological plausibility of PRP in reproductive medicine is based on its pro-angiogenic activity. In PRP, platelets release growth factors, such as VEGF-A, Ang-1, and HGF, that act synergistically to initiate neovascularization, both in case of Ashermans’ syndrome and thin endometrium in women with RIF (29, 38). These signals promote CD31 + endothelial cell proliferation and remodeling of the spiral arteries (29, 53). Hypoxia-inducible factors HIF1*α* and HIF2*α* are thought to play a crucial role in this remodeling process and restore impaired uterine environments (29). This cellular transformation re-establishes vascular stability and microcirculation to reverse uterine hypoxia and oxidative stress in patients with repeated implantation failure (29, 53).

The alteration of cellular parameters leads to an improved uterine hemodynamic profile after PRP exposure. Studies have shown a significant decrease in the RI and PI in the uterine, arcuate, and spiral arteries (38). Traditional assessment of endometrial receptivity is based on endometrial thickness, but recently it was suggest that the presence of multifocal signals—after 2D and color Doppler ultrasound—rather than the endometrial thickness itself, may be a more reliable predictor of receptivity in these patients (54). In women with absent baseline signal blood flow, subendometrial PRP injection may be preferred as a clinical option since it improves local microcirculation and could lead to positive pregnancy outcomes (55).

### 5.3 Immunomodulation and anti-inflammatory effects

Successful implantation depends on the restoration of an optimized uterine microecology, also called the ‘Iron Triangle’ by maintaining the physical endometrium, local immune system, and microbiota (46). Experimental studies have shown that PRP can act as a bioregulator to restore this equilibrium by inducing the shift from pro-inflammatory environments toward a Th2-type profile with increased levels of anti-inflammatory cytokines, such as IL-4 and IL-10 (56, 57). The fetal-maternal tolerance is additionally established through the CD39/CD73 enzymatic pathway on decidual NK cells, which in turn degrade extracellular ATP into adenosine and limit unnecessary activation and inflammation, that are often encountered in patients with RIF (45). To the same direction, experimental data supports PRP-derived Hepatocyte Growth Factor's (HGF) central role in down-regulating pro-inflammatory genes including COX-2, IL-6 and IL-8 (56).

In addition, PRP can reverse dysregulated hormone-microRNA axes, among others by normalizing miR-181 levels, thus contributing to embryo attachment (58). Therefore, PRP might play an important role in reconstructing the ’soil’ for maternal-fetal tolerance.

Finally, PRP seems to inhibit inflammatory cell-populations’ recruitment (56, 59) restraining the pro-inflammatory profile, the latter being considered as hostile for embryo implantation.

### 5.4 Inhibition of fibrosis and tissue remodeling

The regenerative potential of Platelet-rich Plasma is dependent on its ability to act as a biological regulator of the extra-cellular matrix (ECM). This biological regulation induces the shift from the fibrotic uterine state to a functional remodeling state, by modulating the expression of multiple anabolic and catabolic factors (60). *In vitro* studies have demonstrated that platelet-rich plasma stimulates the synthesis of matrix metalloproteinases (MMPs), specifically MMP1, MMP3, MMP7 and MMP26 which degrade the excess accumulation of proteins, therefore facilitating tissue reorganization (9, 61, 62). To the same direction, it has been shown on an ­­animal model that PRP may down-regulate tissue inhibitors of metalloproteinase 1 (TIMP1) (63). These combined propose PRP as a regulator of ECM remodeling in the aim to stabilize the endometrial milieu (9). This carefully regulated cellular and molecular cascade facilitates the regeneration of the ECM, allowing for the reversion from chronic scarring, a result of chronic inflammation often observed in patients with repeated implantation failure.

For patients suffering from Asherman's syndrome, the regenerative potential of Platelet-rich Plasma is dependent on inhibiting the aberrant collagen deposition. In a murine model PRP significantly downregulated fibrosis markers, like COL1A1, ultimately reversing the epithelial lining disruption and gland loss that is observed in fibrotic scars (29, 61). The end result is both myofibroblast differentiation and collagen maturation without the excessive scar deposition that is a typical response to surgical or inflammatory challenges (9). The restoration of the anatomical integrity of the endometrium is driven by the stimulation of stromal fibroblasts and mesenchymal stem cells (MSCs) in the basal layer of the endometrium (61, 62). In addition, Platelet-rich Plasma enhances microecological health by inhibiting NF-kB, a fact which diminishes the chronic inflammation that promotes fibrogenesis (46, 60). By stimulating the endometrium through multiple molecular and cellular cascades, Platelet-rich Plasma returns the endometrium from the pathological state to a receptive state that is able to sustain the embryo implantation (29).

## 6 PRP application in RIF management: evidence from an array of meta-analyses

### 6.1 Reproductive outcomes

After thorough research of the PubMed and Google Scholar databases, 17 meta-analyses were retrieved (14, 64–79). Fourteen of them were systematic reviews with meta-analyses and 3 were systematic reviews with network meta-analyses. Synthesis of the 17 meta-analyses demonstrates that intrauterine infusion of autologous platelet-rich plasma (PRP) suggests a possible benefit both on clinical pregnancy rates (CPR) and live birth rates (LBR) in women undergoing assisted reproduction. Network meta-analyses consistently rank PRP as the most effective intrauterine intervention for enhancing these outcomes when compared to other treatments such as granulocyte colony-stimulating factor (G-CSF), peripheral blood mononuclear cells (PBMCs), and human chorionic gonadotropin (HCG) (67, 69, 79). These benefits were observed across diverse patient populations, specifically those diagnosed with recurrent implantation failure (RIF) and thin endometrium (71, 73–76). Grouped findings suggest that the treatment effect is particularly robust in blastocyst-stage embryo transfers, being in favor for higher CPR compared to cleavage-stage transfers (70, 75). Across these 17 reports, the total sample sizes ranged from 625 to 3,308 participants, encompassing between 7 and 56 primary studies. Statistical heterogeneity for clinical pregnancy rates was found ranging from low to moderate substantial (I2 = 0% to 88%). Conversely, live birth rate analyses frequently exhibited substantial initial heterogeneity (I2 = 40% to 85%), which often resolved in sensitivity analyses after excluding specific large trials or studies with stringent inclusion criteria (Table 1).

**Table 1:** Summary of reproductive outcomes from meta-analyses. Overall results are presented. In case of meta-analyses including more pathological entities including RIF, where available, subgroup analyses on RIF are also presented. (RIF: repeated implantation failure, RPL: recurrent pregnancy loss, TE: thin endometrium, IVF: *in vitro* fertilization, IF: implantation failure).

#	Authors/Article details	PMID	Year	Inclusion criteria	Studies	Patients	CPR	LBR
1.	Maleki-Hajiagha A. et al, J Reprod Immunol 2020 Feb:137:103078 (74)	32006776	2020	IVF (Subgroup analysis for RIF, TE)	7/5 for RIF	625/478 for RIF	RR: 1.79, significant (heterogeneity I^2^ = 16%) RR_RIF_: 1.73, significant (heterogeneity I^2^ = 36%)	N/A
2.	Liu K et al, J Obstet Gynaecol Res 2022 Dec;48 (12):3137–3151 (71)	36104948	2022	IF (Subgroup analysis for RIF, IF < 3)	8/5 for RIF	1219/991 for RIF	OR: 2.24, significant (heterogeneity I^2^ = 61%) OR_RIF_: 2.55, significant (heterogeneity I^2^ = 69%)	
					4/2 for RIF	791/711 for RIF		OR: 5.76, significant (heterogeneity I^2^ = 85%) OR_RIF_: 5.07, significant (heterogeneity I^2^ = 92%)
3.	Hu S et al, Geburtshilfe Frauenheilkd 2022 Dec 13;83 (4):453–462 (65)	37034414	2022	IVF (Subgroup analysis for RIF, TE, RPL)	7/4 for RIF	861/678 for RIF	RR: 2.51, significant (heterogeneity I^2^ = 0%) RR_RIF_: 2.46, significant (heterogeneity I^2^ = 0%)	
					2	433		RR: 7.03, significant (heterogeneity I^2^ = not examined) RR_RIF_: not examined
4.	Kong X et al, J Reprod Immunol 2023 Mar:156:103819 *Network Meta-analysis* (69)	36774717	2023	RIF	21	2917	OR: 0.28, significant (PRP vs. Blank) OR: 0.41, significant (PRP vs. Placebo) *Significance of PRP administration is towards 0.*	OR: 0.15, significant (PRP vs. Blank) OR: 0.27, significant (PRP vs. Placebo) *Significance of PRP administration is towards 0.*
5.	Maged AM et al, J Assist Reprod Genet 2023 May;40 (5):969–983. (73)	37010710	2023	IF, IVF, TE (Subgroup analysis for RIF, IF TE)	12 (RCTs) 6 (RCTs_RIF_)	1374 (RCTs) 501 (RCTs_RIF_)	RCTs. OR: 2.21, significant (heterogeneity I^2^ = 88%) RCTs_RIF_. OR: 1.95, significant (heterogeneity I^2^ = 47%)	
					10 (Non-RCTs)	1452 (Non-RCTs)	Non-RCTs. OR: 2.39, significant (heterogeneity I^2^ = 68%)	
					4 (RCTs) 2 (RCTs_RIF_)	523 (RCTs) 130 (RCTs_RIF_)		RCTs. OR: 4.35, non significant, (heterogeneity I^2^ = 84%) RCTs_RIF_. OR: 2.36, non significant, (heterogeneity I^2^ = 68%)
					10 (Non-RCTs)	1452 (Non-RCTs)		Non-RCTs. OR: 4.18, significant, (heterogeneity I^2^ = 65%)
6.	Shalma NM et al, BMC Pregnancy Childbirth 2023 Dec 8;23 (1):843 (76)	38066425	2023	IVF (Subgroup analysis for RIF, TE)	20/13 for RIF	2166/1772 for RIF	RR:1,84 significant (heterogeneity I^2^ = 29%) RR_RIF_:1,83 significant (heterogeneity I^2^ = 43%)	
					10/6 for RIF	1209/1003 for RIF		RR: 2.31, significant (heterogeneity I^2^ = 68%). After omitting a study, RR: 1.75, significant (heterogeneity I^2^ = 15%) RR_RIF_: 2.54, significant (heterogeneity I^2^ = 76%). After omitting a study, RR_RIF_: 1.83, significant (heterogeneity I^2^ = 5%)
7.	Deng H et al, J Obstet Gynaecol 2023 Dec;43 (1):2144177 (14)	36397660	2023	RIF	10	1555	RR: 1.96, significant (heterogeneity I^2^ = 46%) After omitting a study, RR: 2.09, significant (heterogeneity I^2^ = 0%)	RR: 2.83, significant, (heterogeneity I^2^ = 83%) After omitting a study, RR: 1.93, significant (heterogeneity I^2^ = 0%)
8.	Huang C et al, Iran J Public Health 2023 Aug;52 (8):1542–1554 (66)	37744529	2023	IVF	14	1081	OR: 2.34, significant (heterogeneity I^2^ = 27%)	
9.	Soliman A et al, Turk J Obstet Gynecol 2023 Jun 1;20 (2):154–163. (77)	37260250	2023	RIF	8	981	RR: 2.35, significant (heterogeneity I^2^ = 0%)	
					3	553		RR: 4.03, significant (heterogeneity I^2^ = 83%) After omitting a study, RR: 7.03, significant (heterogeneity I^2^ = 0%)
10.	Xie Q et al, Reprod Biol Endocrinol 2024 Apr 16;22 (1):44. *Network Meta-analysis*. (79)	38627790	2024	RIF	13	1704	OR: 2.90, significant (heterogeneity I^2^ = 32%)	
					4	641		OR: 4.27, significant (heterogeneity I^2^ = 79%)
11.	Kaur H et al, J Hum Reprod Sci 2024 Jan-Mar;17 (1):2–15 (68)	38665609	2024	RIF	8	1018	OR: 3.20, significant (heterogeneity I^2^ = 0%)	
					4	633		OR: 7.32, significant (heterogeneity I^2^ = 40%)
12.	Vaidakis D et al, Cochrane Database Syst Rev 2024 Apr 29;4 (4):CD013875 (78)	38682756	2024	IVF	9	824	OR: 2,22, significant, GRADE: very low	
					6	564		OR: 2.38, significant GRADE: very low
13.	Ma T and Pu Y, Clin. Exp. Obstet. Gynecol. 2024; 51 (2): 48 (72)	N/A	2024	RIF	15	1961	RR: 1.78, significant (heterogeneity I^2^ = 26%)	
					4	811		RR: 2.62, significant (heterogeneity I^2^ = 87%)
14.	Alkhamis FH, Int J Community Med Public Health. 2024 Nov;11 (11):4423–4431 (64)	N/A	2024	IVF	19	1918	OR: 2.49, significant (heterogeneity I^2^ = 43%)	
					6	777		OR: 3.28, significant (heterogeneity I^2^ = 77%)
15.	Jiang L et al, J Assist Reprod Genet 2025 Apr;42 (4):1177–1190*. Network Meta-analysis*. (67)	40035946	2025	RIF	25	3035	OR: 0.33, significant (PRP vs. Blank) OR: 0.53, significant (PRP vs. Placebo) *Significance of PRP administration is towards 0.*	OR: 0.27, significant (PRP vs. Blank) OR: 0.54, significant (PRP vs. Placebo) *Significance of PRP administration is towards 0.*
16.	Pan Z et al, Front Endocrinol (Lausanne) 2026 Apr 14:17:1730259 (75)	42058785	2026	RIF	10	1208	OR: 3.18, significant (heterogeneity I^2^ = 3%)	
					5	756		OR: 5.10, significant (heterogeneity I^2^ = 75%)
17.	Liang X et al, Reprod Med Biol 2026 Jul 6;25 (1):e70070 (70)	42440797	2026	RIF	10	1188	RR: 2.23, significant (heterogeneity I^2^ = 0%)	
					5	756		RR: 3.74, significant, (heterogeneity I^2^ = 79%)

Despite these positive trends, major contradictions exist regarding the population most likely to benefit; individual meta-analyses reported that PRP did not significantly improve outcomes for non-RIF patients or those who had experienced fewer than three implantation failures (71, 73, 75). Furthermore, some meta-analyses found no statistically significant reduction in miscarriage risk, while others reported favorable outcomes. Despite the positive results, certainty is tempered by the “low” to “very low” quality of evidence cited in several reviews, attributed to small trial sample sizes and high risks of performance bias. This reduces confidence in PRP as a contemporary approach, a fact noted in ESHRE guidelines for RIF management (8).

### 6.2 Critical appraisal

A critical evaluation of the current evidence base for Platelet-rich Plasma (PRP) in repeated implantation failure (RIF) reveals significant methodological deficiencies. As recently reported (3), the application of the Cochrane risk-of-bias tool (RoB 2) indicates that every available randomized controlled trial (RCT) in this field suffers from a high overall risk of bias. These systemic failures are particularly evident in the randomization processes and the inadequate management of missing outcome data, which severely compromise the internal validity of the findings (3). Trustworthiness is further undermined by a notable lack of trial registration and frequent discrepancies between registered protocols and the final published manuscripts (3). Consequently, meta-analyses that pool these low-quality primary studies may inadvertently compound methodological errors, leading to misleadingly positive clinical results that do not reflect true therapeutic efficacy (3).

The lack of effective blinding represents a major source of performance bias in RIF research. Methodological scrutiny highlights that many primary RCTs failed to blind either participants or outcome assessors, which is a critical requirement for objective evaluation (73). In many studies, the control group received no intervention, making it difficult to isolate the biological effects of PRP from the mechanical trauma of the procedure itself (73). This “catheterization effect” may simulate endometrial injury, similar to endometrial scratching, thereby confounding the observed results (73). Without the implementation of robust, sham-controlled trials, the reported statistical gains in clinical pregnancy rates remain susceptible to significant detection and performance bias, casting doubt on whether the improvements are truly biological in nature (73).

A fundamental issue lies in the definition of the study population; none of the current trials utilize RIF definitions that meet the minimum evidence-based criteria proposed by ESHRE (3). This diagnostic heterogeneity likely leads to significant misclassification, with false-positive RIF diagnosis rates estimated at 46% after only two failed cycles (80). It has been noted that many couples are inappropriately labeled with RIF, potentially inflating the success rates of experimental adjuvants (80). In contrast with this notion, a recent meta-analysis highlights that the positive PRP effect on reproductive outcomes refers to cases with at least three failed attempts, whereas less than three attempts are not to benefit from the intervention (75). The role of age as a confounding factor has been rather frequently ignored. It has been reported that in case of thin endometrium in women with age greater than 35 years, PRP administration fails to improve live birth rates; this failure has not been observed in women being younger than 35 years (76). However, the evidence is rather weak for both the number of previous failed attempts and women's age to be considered as established criteria in selecting candidates for PRP administration.

GRADE assessments, wherever available, categorize the overall quality of evidence for live birth and pregnancy rates as low or very low, underscoring the need for more rigorous standardization before PRP can be considered a proven standard of care (73). To this direction, the recent Cochrane meta-analysis on PRP application in human reproduction highlights that although a positive impact of PRP on reproductive outcomes has been reported, there is substantial uncertainty about the real impact of PRP on human reproduction since a series of limitations significantly reduces the quality of the included studies (78). In line with the overall uncertainty depicted, is also a recent umbrella review in which relevant systematic reviews and meta-analyses were analyzed. The lack of GRADE assessment is noted to the majority of the included studies, along with a significant level of overlapping due to the inclusion of the same primary evidence; these observations reduce the strength of the produced evidence thereafter advising for caution in applying PRP as a means of improving reproductive outcomes in contemporary practice (81).

The evidence weakens further by the lack of reporting of the protocol of PRP preparation; only 10% of the studies provide a fully detailed description of their methods (15). It has been shown that number of centrifugations (single vs. double) and the mode of PRP activation (mechanical or chemical) are directly affecting the time-sensitive release of growth factors (19, 36, 37). The final concentration of platelets along with the volume of PRP infused and number of infusions per cycle are also varying between the studies (15, 82). Only recently it was shown that 0.5 mL of PRP were not inferior to greater doses in terms of outcomes (70). To the same direction, the time-point of the infusion seems to alter the outcomes. Several studies report PRP being infused 48 h prior embryo-transfer while others perform PRP administration at earlier time-points (73). Interestingly, it was shown that infusing PRP at earlier time-points jeopardizes clinical outcomes (77). The profound lack of consensus in PRP preparation and administration can justify the heterogeneity among the studies and the urgent need for a standardized formula that incorporates PRP volume and platelet concentration compared to baseline blood parameters.

Finally, although PRP is considered safe in the short run as an autologous procedure, there is no evidence regarding a potential long-term impact on the perinatal and the neonatal outcomes. Therefore, even if proven beneficial, PRP administration may be employed with caution and after proper discussion with the patients.

## 7 Conclusion

This Review Paper concludes at the crossroads where high biological plausibility meets low clinical certainty. The secretome of PRP contains a complex array of growth factors such as VEGF and PDGF that, at a cellular level, support tissue repair and angiogenesis. Moreover, PRP seems to induce immunomodulation towards maternal-fetal tolerance. The data produced from the experimental setting have triggered great enthusiasm leading to consider PRP as a promising add-on, especially in case of RIF. In this context, several RCTs and non-RCTs have been published, highlighting a potential role of PRP on reproductive outcomes. To the same direction, the meta-analyses that included these trials demonstrated a tentative trend for PRP application in assisted reproduction interventions. Interestingly, a careful examination of the evidence has revealed several levels of bias. Heterogeneity is stemming from the lack of standardization in PRP preparation, combined with the uncertainty upon patient selection and inclusion due to different and constantly updated RIF definitions. In this context, PRP applications have not been incorporated in the ESHRE guidelines for contemporary practice. Thus, PRP should only be used within the frame of clinical trials provided that patients have been properly informed. To the best of our knowledge our work is one of the efforts aiming to present a targeted review balancing between the strong biological and the weak clinical evidence. We intended to highlight this difference in order to trigger proper discussions for proper designing of relevant RCTs.
